# A Case of Giant Bladder Carcinosarcoma without Submucosal Invasion

**DOI:** 10.1155/2011/349518

**Published:** 2011-05-05

**Authors:** Masayoshi Zaitsu, Mariko Yamanoi, Koji Mikami, Akiko Tonooka, Toshimasa Uekusa, Takumi Takeuchi

**Affiliations:** ^1^Department of Urology, Kanto Rosai Hospital, 1-1 Kizukisumiyoshi-Cho, Nakahara-ku, Kawasaki 211-8510, Japan; ^2^Department of Pathology, Kanto Rosai Hospital, 1-1 Kizukisumiyoshi-Cho, Nakahara-ku, Kawasaki 211-8510, Japan

## Abstract

Carcinosarcoma is a rare biphasic neoplasia containing both malignant mesenchymal and epithelial elements. Bladder carcinosarcoma commonly presented as high-grade, advanced stage, and aggressive behavior with a poor prognosis. An 83-year-old male presented with painless gross hematuria to our hospital. Cystoscopy revealed massive nonpapillary bladder tumor on the right wall. The 91 g tumor could be completely removed with transurethral resection. Histology of the tumor was diagnosed as carcinosarcoma with no submucosal invasion composed of biphasic malignant epithelial and mesenchymal cells. Epithelial malignancy was urothelial cancer and mesenchymal one was chondrosarcoma and leiomyosarcoma. The specimens taken at the second-look TUR-Bt revealed that carcinoma in situ (urothelial cancer) but not sarcoma existed at the mucosa surrounding the previous tumor site. 80 mg of BCG instillation intravesically every week for six weeks was successfully administered to the patient. There is no tumor recurrence for 6 months after treatments.

## 1. Introduction

Carcinosarcoma, also called true malignant mixed tumor, is a rare biphasic neoplasia containing both malignant mesenchymal and epithelial elements. Only 79 cases of carcinosarcoma of bladder origin have been reported in the English literature in the year of 2007 [1]. Bladder carcinosarcoma commonly presented as high-grade, advanced stage, and aggressive behavior with a poor prognosis [2]. 

The mesenchymal element of carcinosarcoma lacks epithelial markers [3, 4] and patients with carcinosarcoma present at a more advanced stage and are at greater risk for death compared to patients with high-grade urothelial carcinoma [5]. In a clinicopathological report analyzing 15 cases of bladder carcinosarcoma, chondrosarcoma in 3 cases, leiomyosarcoma in 3, malignant fibrous histiocytoma in 3, osteosarcoma in 2, fibrosarcoma in 1, rhabdomyosarcoma in 1, and more than 1 type in 2 were demonstrated in the sarcomatous component [3].

In cases of bladder carcinosarcoma, evidence supporting a monoclonal origin for the epithelial and mesenchymal components was revealed using loss of heterozygosity studies with microsatellite markers [6, 7], while there exists a hypothesis that multiclonal stem cells of the epithelial and mesenchymal components play a causative role. It is also known that bladder carcinosarcoma develops following cyclophosphamide therapy [6, 8] as well as radiotherapy to the bladder [9]. Here we report an interesting case of bladder carcinosarcoma which was more than 10 cm in diameter, nevertheless it was without submucosal invasion.

## 2. Case Presentation

**Figure 1:**
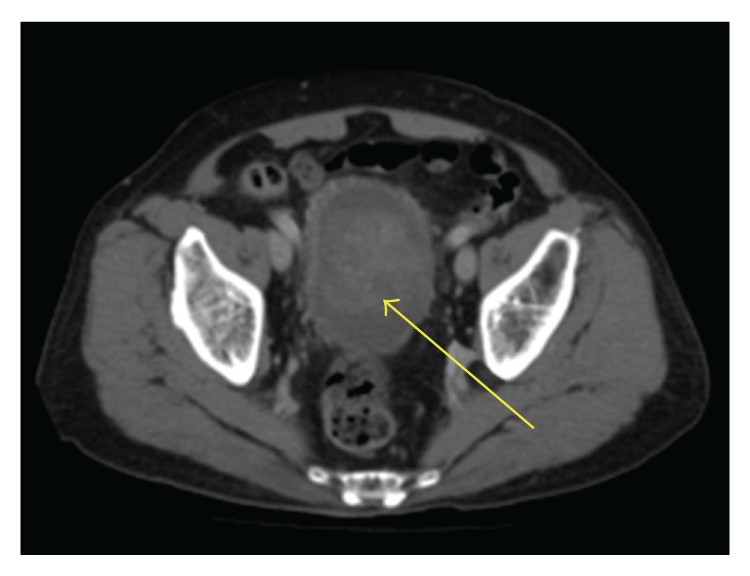
Enhanced CT scan indicates a giant bladder tumor (hematoxylin and eosin stain; an arrow indicates the tumor).

An 83-year-old male presented with painless gross hematuria to our hospital.Cystoscopy revealed massive nonpapillary bladder tumor on the right wall. Pelvic CT scan showed a giant bladder tumor more than 10 cm in diameter (Figure 1), while there was no distant metastasis by abdominal and chest CT scans. There were no abnormalities in regular laboratory tests. Fortunately, the tumor could be completely removed with transurethral resection (TUR-Bt) and the tumor base specimen was sampled with cold punch biopsies. The resected tumor weight was 91 g and blood transfusion was unnecessary.

**Figure 2:**
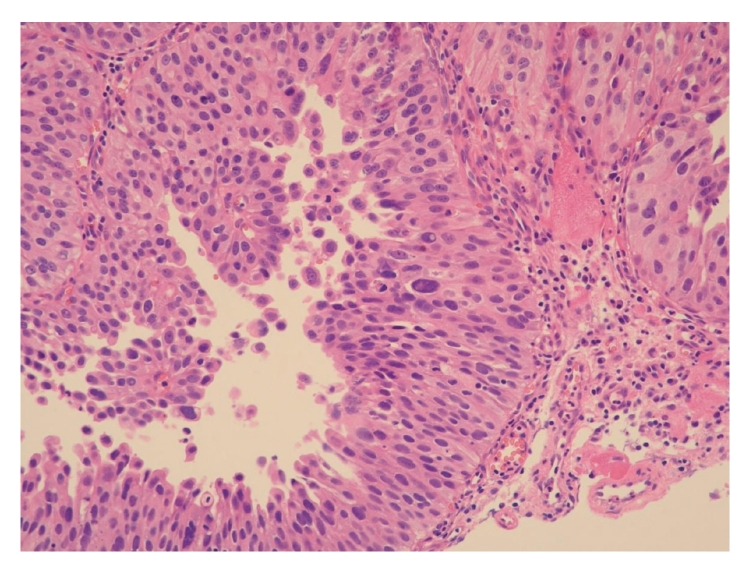
Epithelial malignancy is urothelial cancer without submucosal invasion (hematoxylin and eosin stain).

**Figure 3:**
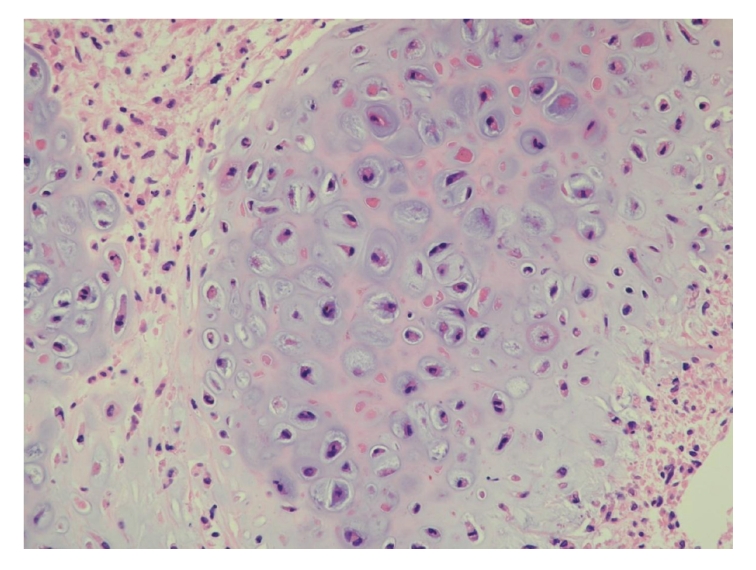
One of the mesenchymal malignancies is chondrosarcoma (hematoxylin and eosin stain).

**Figure 4:**
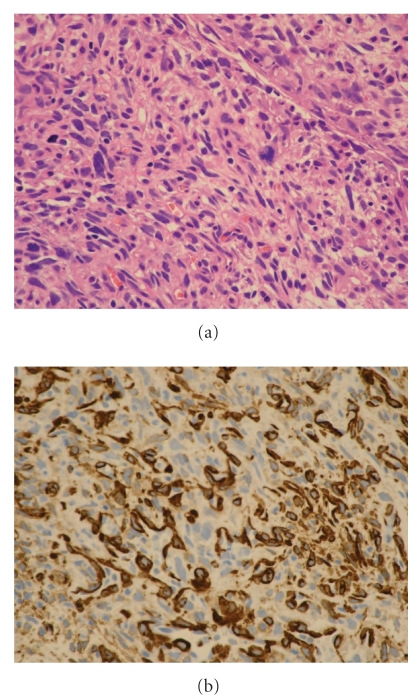
(a) The other of the mesenchymal malignancies is leiomyosarcoma (Hematoxylin and eosin stain). (b) Leiomyosarcoma expressed smooth muscle actin (Immunohistochemistry).

Histology of the tumor was diagnosed as carcinosarcoma composed of biphasic malignant epithelial and mesenchymal cells. Epithelial malignancy was urothelial cancer (Figure 2) and mesenchymal one was chondrosarcoma (Figure 3) and leiomyosarcoma (Figures 4(a) and 4(b)). Broad neck of the tumor was of urothelial cancer grade 2 > 3, and there was no submucosal invasion (Figure 2). Specimen of the tumor base contained muscle fibers and showed no malignant cells there. Immunohistochemistry showed that cytokeratin was positive only in epithelial malignancy but not in mesenchymal one (data not shown).

Considering the noninvasive pathology of the tumor despite well-known aggressive behavior of carcinosarcoma, it was difficult to judge if more invasive treatments such as radical cystectomy or external beam radiation therapy + interventional arterial infusion of anticancer drug(s) are mandatory. Thus, second-look TUR-Bt was done 7 weeks following the first TUR-Bt. The specimens taken at the second-look TUR-Bt revealed that there was no remaining tumor cells at the site where the previous tumor was resected by the first TUR-Bt, while carcinoma in situ (urothelial cancer) but not sarcoma existed at the mucosa surrounding the previous tumor site (data not shown). Then, we forwarded to the intravesical BCG instillation therapy to the patient and 80 mg of BCG every week for six weeks was successfully administered. Pelvic, abdominal, and chest CT scans as well as cystoscopy and urine cytology have not shown tumor recurrence for 6 months after treatments.

## 3. Discussion

A case of bladder sarcomatoid carcinoma, which was resected by TUR-Bt and diagnosed as nonmuscle invasive (pT1 stage), was previously reported to have shown intravesical recurrence during followup, undergone partial and total cystectomy, and finally died 13 months after diagnosis [10]. On the contrary, there was a case of bladder carcinosarcoma which obtained pathologically complete response by chemoradiotherapy [11].

In general, more aggressive therapies than TUR-Bt alone are usually adopted as treatments for bladder carcinosarcoma due to invasiveness of the tumor. The pT stage of our case was fortunately pTa without submucosal infiltration if we simply apply the pathological staging system of bladder urothelial cancer and the second-look TUR-Bt revealed carcinoma in situ but not already invasive cancer/sarcoma, then we did not perform radical cystectomy or chemoradiotherapy in our case, but intravesical BCG instillation therapy trying to preserve bladder. It might have been fortunate that the tumor base was mainly composed of urothelial cancer but not sarcoma, as the latter may often be more invasive than the former. Of course, the patient naturally needs close followup for tumor local recurrence and metastasis. CT scans, cystoscopy, and urine cytology every three months may be necessary during the first year after treatment.
